# TCPL: task-conditioned prompt learning for few-shot cross-subject motor imagery EEG decoding

**DOI:** 10.3389/fnins.2025.1689286

**Published:** 2025-11-24

**Authors:** Pengpai Wang, Tiantian Xie, Yueying Zhou, Peiliang Gong, Rosa H. M. Chan

**Affiliations:** 1 College of Computer and Information Engineering, Nanjing Tech University, Nanjing, China; 2 City University of Hong Kong Shenzhen Research Institute, Shenzhen, China; 3 State Key Laboratory of Terahertz and Millimeter Waves, City University of Hong Kong, Hong Kong, China; 4 Department of Electrical Engineering, City University of Hong Kong, Hong Kong, China; 5 School of Mathematics Science, Liaocheng University, Liaocheng, China; 6 College of Artificial Intelligence, Nanjing University of Aeronautics and Astronautics, Nanjing, China

**Keywords:** motor imagery, EEG decoding, task-conditioned prompt, few-shot learning, transformer, meta-learning

## Abstract

Motor imagery (MI) electroencephalogram (EEG) decoding plays a critical role in brain–computer interfaces but remains challenging due to large inter-subject variability and limited training data. Existing approaches often struggle with few-shot cross-subject adaptation, as they require either extensive fine-tuning or fail to capture individualized neural dynamics. To address this issue, we propose a Task-Conditioned Prompt Learning (TCPL), which integrates a Task-Conditioned Prompt (TCP) module with a hybrid Temporal Convolutional Network (TCN) and Transformer backbone under a meta-learning framework. Specifically, TCP encodes subject-specific variability as prompt tokens, TCN extracts local temporal patterns, Transformer captures global dependencies, and meta-learning enables rapid adaptation with minimal samples. The proposed TCPL model is validated on three widely used public datasets, GigaScience, Physionet, and BCI Competition IV 2a, demonstrating strong generalization and efficient adaptation across unseen subjects. These results highlight the feasibility of TCPL for practical few-shot EEG decoding and its potential to advance the development of personalized brain–computer interface systems.

## 1 Introduction

Motor imagery (MI) electroencephalogram (EEG) decoding has long been a central challenge in brain–computer interface (BCI) research, offering promising applications in motor rehabilitation, assistive communication, and intelligent neuroprosthetics (Lotte et al., 2018; Lu et al., 2021; Craik et al., 2019; Roy et al., 2019). The ability to accurately decode motor intentions directly from neural activity enables seamless interaction between humans and machines, thereby extending motor capabilities and improving the quality of life for individuals with neurological impairments (Lotte, 2020; Lionakis et al., 2023). Although substantial advances have been achieved in recent years, achieving reliable MI decoding remains challenging due to the inherently noisy, non-stationary nature of EEG signals and their considerable inter-subject variability (Jayaram et al., 2016; Kostas and Rudzicz, 2021). Traditional methods based on handcrafted feature extraction, such as common spatial pattern analysis or frequency-domain filtering, often fail to effectively capture the intricate spatiotemporal dependencies inherent in EEG dynamics (Ang et al., 2012; Li, 2019). More recent advances in deep learning have brought substantial improvements by automatically learning multilevel features from raw signals (Schirrmeister et al., 2017; Lawhern et al., 2018; Sun et al., 2022). However, even state-of-the-art deep architectures encounter substantial limitations in real-world BCI deployment, particularly when adapting to unseen subjects with very limited calibration data (Zhou et al., 2023; Wu et al., 2020).

Existing EEG decoding frameworks typically face two major challenges. First, inter-subject variability strongly impacts model generalization (Zhao et al., 2020; Chen et al., 2024). Each subject's brain dynamics are shaped by anatomical differences, electrode placement variations, and diverse cognitive strategies during motor imagery, which collectively cause significant distribution shifts across subjects (Liu, 2020; Kostas and Rudzicz, 2021). Models trained on pooled datasets often underperform when transferred to new individuals, as they fail to capture these subject-specific dynamics (Kwak et al., 2023).

Second, few-shot cross-subject adaptation remains particularly problematic, as it involves adapting to new subjects with only a few labeled trials per class. Many approaches require large volumes of calibration data for each new user, which is impractical in real clinical or daily-life BCI applications (Lu et al., 2021; Zhao et al., 2020). Although transfer learning and domain adaptation techniques have been explored (Wu et al., 2020; Xu et al., 2021; Kwak et al., 2023), they often involve extensive fine-tuning of the entire network or assume domain-invariant feature spaces that may not fully represent individual variability (Li, 2019; Zhou et al., 2023). As a result, current methods either sacrifice efficiency or fail to provide personalized adaptability, limiting their scalability and applicability in real-world BCI systems (Lotte, 2020).

Recent work has also explored more specialized strategies for cross-subject EEG adaptation. For example, meta-transfer and few-shot meta-learning variants aim to learn rapid adaptation rules across subjects (Duan et al., 2020; Zhou et al., 2019), where a meta-task typically corresponds to the support–query split of an individual subject during episodic meta-training; graph-based methods attempt to model channel relationships explicitly for cross-subject alignment; and a growing body of research has investigated conditional or attention-based mechanisms to inject subject- or session-specific context into deep decoders. Notably, recent attention-driven and hybrid CNN–Transformer architectures, such as LMDA-Net (Miao et al., 2023), CNNViT-MILF-a (Zhao et al., 2025), and other attention-enhanced frameworks for motor imagery decoding (Wimpff et al., 2024; Han et al., 2025), further demonstrate the potential of integrating spatial–temporal attention to enhance generalization and interpretability in EEG-based BCI. These lines of work provide closer points of comparison to our approach and motivate the need for parameter-efficient conditioning mechanisms that operate with only a few calibration trials.

To address these shortcomings, we propose Task-Conditioned Prompt Learning (TCPL), a novel framework that introduces the idea of task-conditioned prompts into neural decoding. The term “task” is used in two related senses in this work: (i) at the meta-learning level, a *meta-task* corresponds to a subject-level adaptation problem (i.e., adapting to a particular subject given a small support set); and (ii) at the signal level, a *task* may refer to the MI class distinction (e.g., left vs. right hand). In TCPL, prompt tokens are generated per meta-task (subject) to capture subject-specific priors while the classifier still predicts MI task labels. The central motivation behind TCPL is that EEG inter-subject differences can be treated as implicit task conditions, which can be explicitly modeled as a set of learnable prompt tokens (Liu, 2021; Zhou et al., 2022).

Unlike conventional fine-tuning approaches that adjust network parameters, our method leverages these task-conditioned prompts to encode individualized neural signatures and inject them directly into the feature extraction pipeline. The prompt refers here to a small set of continuous tokens that condition the downstream encoder on subject-specific context. Specifically, TCPL incorporates a Task-Conditioned Prompt (TCP) module that generates subject-specific prompts from a few calibration samples, a Temporal Convolutional Network (TCN) for capturing local temporal dynamics of oscillatory patterns (Bai et al., 2018), and a Transformer module for modeling global cross-channel dependencies (Vaswani et al., 2017; Dosovitskiy et al., 2021). These modules are jointly optimized within a meta-learning framework (Finn et al., 2017; Nichol et al., 2018), which trains the model across multiple subject-level tasks, enabling it to rapidly adapt to new individuals with minimal calibration data (Duan et al., 2020; Zhou et al., 2019). Prompt-based conditioning is especially suitable for EEG because (i) it enables parameter-efficient personalization (only the prompt generator needs to synthesize task-specific tokens), (ii) it lets the backbone retain a stable shared representation while being dynamically modulated by subject context, and (iii) continuous prompts can capture subtle distributional shifts without overfitting on extremely small support sets.

Prompt-based conditioning is particularly suitable for EEG data due to several inherent properties of the signals and the learning framework. First, EEG recordings are typically low in signal-to-noise ratio and exhibit substantial inter-subject and inter-session variability; using prompt tokens allows efficient incorporation of subject-specific priors without modifying the backbone parameters, thereby reducing overfitting. Then, prompts function as adaptable bias vectors that capture distributional differences, such as electrode configurations or baseline spectral patterns, and modulate feature extraction in a personalized manner. By meta-learning prompt generation across multiple subjects, the model can encode common adaptation dynamics, achieving rapid few-shot personalization during testing. Finally, the continuous and composable nature of prompts enables seamless integration with hybrid architectures such as the TCN–Transformer, where prompts provide subject-aware conditioning in the temporal domain, facilitating adaptive feature aggregation and enhancing generalization.

By explicitly modeling inter-subject variability and steering the representation learning process through task-conditioned prompts, TCPL attains efficient and robust few-shot EEG decoding. The proposed TCPL model offers several distinctive features that address the fundamental challenges of MI EEG decoding. First, it pioneers the use of task-conditioned prompts to represent subject individuality, providing a lightweight yet powerful mechanism for cross-subject personalization without retraining the entire network. Second, the hybrid TCN–Transformer backbone effectively unifies local temporal feature extraction with global spatial dependency modeling, ensuring that both short-range oscillatory rhythms and long-range cross-regional interactions are preserved. Third, the meta-learning framework equips TCPL with the ability to generalize across heterogeneous subject populations while retaining the flexibility to adapt rapidly to new users.

In summary, this work makes the following contributions:

1. We introduce TCPL, a novel task-conditioned prompt learning framework for few-shot cross-subject motor imagery EEG decoding, which explicitly encodes subject individuality as prompt tokens for efficient adaptation.
2. We design a hybrid backbone, combining Temporal Convolutional Networks and Transformer layers, to jointly capture local temporal dynamics and global spatial dependencies in EEG signals.
3. We embed TCPL within a meta-learning paradigm, enabling rapid adaptation to new subjects with only a few calibration samples while ensuring robust cross-subject generalization.
4. We conduct extensive evaluations on two widely used public datasets, demonstrating that TCPL consistently outperforms state-of-the-art baselines in few-shot cross-subject settings.

## 2 Methodology

**Figure 1:**
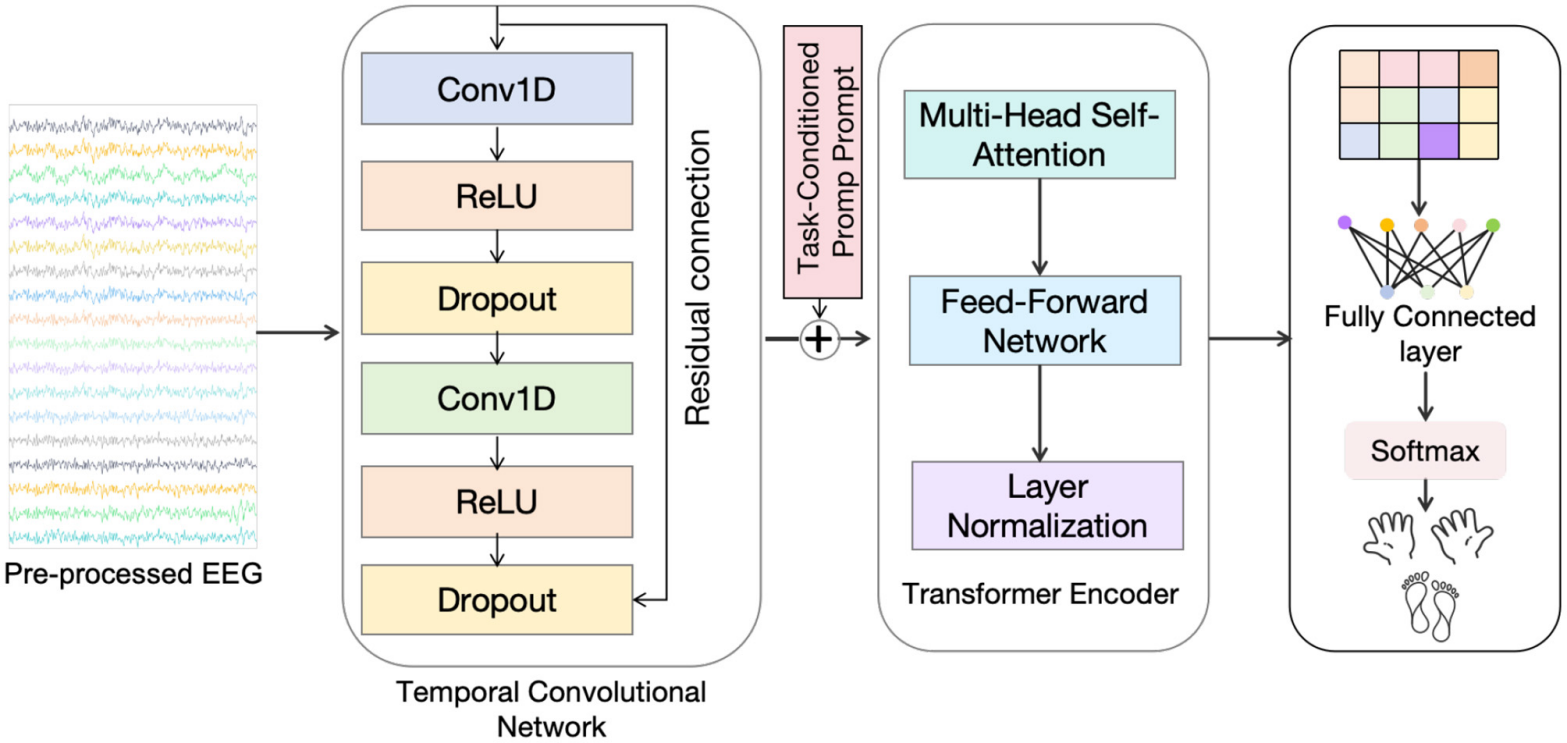
The architecture of the proposed TCPL model. It consists of a Task-Conditioned Prompt (TCP) module that generates subject-specific prompts from a few-shot support set, a Temporal Convolutional Network (TCN) backbone for local temporal feature extraction, and a Transformer module that integrates prompts with sequence features to capture global dependencies for robust few-shot EEG decoding.

In this section, we present the TCPL (Task-Conditioned Prompt Learning) model for few-shot cross-subject motor imagery EEG decoding (Figure 1). We first provide an overview of the model architecture and then describe the key components, including the Task-Conditioned Prompt (TCP) module, the Temporal Convolutional Network (TCN), the Transformer module, and the meta-learning framework. Finally, we detail the training objectives and the inference procedure.

### 2.1 Overview of TCPL

The proposed Task-Conditioned Prompt Learning (TCPL) framework is designed to achieve efficient and robust cross-subject EEG decoding by explicitly modeling subject-specific variability as learnable prompt tokens. Unlike conventional fine-tuning or domain adaptation methods that require extensive retraining, TCPL introduces a lightweight mechanism that rapidly personalizes the decoding process to new subjects with only a few calibration trials. The core idea is to treat inter-subject differences as implicit “tasks,” and encode these differences through task-conditioned prompts that adapt the model's feature extraction and attention mechanisms without modifying the backbone parameters. The framework consists of three tightly integrated components: (1) a **Task-Conditioned Prompt (TCP) module** that generates a compact set of prompt tokens representing the neural signature of a given subject, based on a few labeled samples; (2) a **Temporal Convolutional Network (TCN)** that efficiently captures hierarchical temporal dynamics and local oscillatory patterns from raw EEG signals; and (3) a **Transformer encoder** that models global cross-channel dependencies, where the generated prompts are injected into the self-attention layers to modulate attention weights in a subject-specific manner. These components are jointly trained under a meta-learning framework that simulates few-shot adaptation across subjects during training, thereby allowing the model to generalize quickly to unseen individuals. Through this design, TCPL bridges the gap between personalized calibration and generalizable feature learning, enabling fast, adaptive, and reproducible EEG decoding for real-world brain–computer interface applications.

### 2.2 Task-conditioned prompt module

The TCP module is designed to encode subject-specific variability and provide individualized guidance to the feature extractor. Given a support set $S={\left\{\left(X_{i},y_{i}\right)\right\}}_{i=1}^{N_{\text{shot}}}$ from a new subject, the TCP generates a set of prompt tokens:


$$\begin{array}{l} P=g_{\phi }\left(S\right), \end{array}$$ (1)

where *g*_ϕ_ is a small neural network parameterized by ϕ that maps the few-shot samples to *L* prompt tokens *P* = {*p*_1_, …, *p*_*L*_}, with each $p_{l}\in {\mathbb{R}}^{d}$.

We explicitly specify the encoder, pooling, and prompt-generator used in our implementation. Let each trial $X_{i}\in {\mathbb{R}}^{C\times T}$ be first processed by a lightweight support encoder *f*_ϕ_(·) (shared across tasks) that maps a trial to a *d*-dimensional embedding:


$$\begin{array}{l} e_{i}=f_{\phi }\left(X_{i}\right)\in {\mathbb{R}}^{d}. \end{array}$$ (2)

In practice *f*_ϕ_ consists of two 1-D convolutional layers (kernel size 3, stride 1, padding same), followed by global average pooling across time and a linear projection to dimension *d*. We compute a summary subject embedding by mean pooling over support embeddings:


$$\begin{array}{l} h_{s}=\frac{1}{N_{\text{shot}}}\overset{N_{\text{shot}}}{\underset{i=1}{\sum }}e_{i}\in {\mathbb{R}}^{d}. \end{array}$$ (3)

The prompt generator *g*_ψ_ (notation changed to ψ to distinguish generator params) is implemented as a two-layer MLP with hidden width *d* and ReLU activation that maps *h*_*s*_ to *k* outputs, reshaped into *k* prompt tokens:


$$\begin{array}{l} P_{s}=g_{\psi }\left(h_{s}\right)=\left[p_{s,1};\ldots ;p_{s,k}\right]\in {\mathbb{R}}^{k\times d}. \end{array}$$ (4)

We use *k* = 10 and *d* = 64 as default values. Prompts are initialized from N(0,0.01) and are not independent free parameters—they are deterministic outputs of *g*_ψ_(*h*_*s*_) and therefore updated only via ψ during meta-training.

In the original text prompt concatenation was described along the channel dimension. For clarity and reproducibility, we explicitly state the approach used in experiments: after TCN processing (see next subsection) the temporal feature sequence is *H*∈ℝ^*T*^′ × *d*. We concatenate prompts as prefix tokens along the temporal (sequence) dimension, forming the Transformer input


$$Z=\left[P_{s};H\right]\in {\mathbb{R}}^{\left(k+T^{\prime }\right)\times d}.$$

This choice ensures direct interaction of prompts with sequence tokens via self-attention.

For conceptual continuity, the channel-wise concatenation $\tilde{X}=\left[X;P\right]$ is shown here, but in our implementation, prompt tokens are never concatenated to the raw EEG input. Instead, they are appended as temporal prefix tokens after TCN feature extraction and before the Transformer:


$$Z=\left[P_{s};H\right]\in {\mathbb{R}}^{\left(k+T^{\prime }\right)\times d},$$

where *H*∈ℝ^*T*^′ × *d* is the TCN output sequence. This ensures that the prompts guide the Transformer via sequence-based self-attention while leaving TCN feature extraction on raw EEG unaltered.

For reproducibility we replace the above $\tilde{X}$ usage in the rest of the Method section by the sequence-based notation *Z* = [*P*_*s*_; *H*] once TCN features *H* are available. The equation above is retained for conceptual continuity with earlier descriptions, and the precise implementation used in experiments is (*Z* = [*P*_*s*_; *H*]) as explained.

In our TCPL implementation, prompt tokens are never concatenated to raw EEG channels. Instead, they are always appended along the temporal (sequence) dimension after TCN feature extraction and before the Transformer. This consistent sequence-based injection ensures that subject-specific prompts modulate the Transformer attention while keeping TCN feature extraction unaltered.

### 2.3 Temporal convolutional network module

The TCN module extracts local temporal features from EEG signals. We use causal convolutions with dilated kernels to model long-range dependencies efficiently. For each layer *l*, the TCN operation is defined as:


$$\begin{array}{l} h^{\left(l\right)}=\sigma \left(W^{\left(l\right)}*h^{\left(l-1\right)}+b^{\left(l\right)}\right), \end{array}$$ (5)

where * denotes the causal dilated convolution, $h^{\left(0\right)}=\tilde{X}$, *W*^(*l*)^, and *b*^(*l*)^ are learnable weights and biases, and σ is an activation function (e.g., ReLU). The receptive field grows exponentially with the number of layers due to dilation, allowing the network to capture both short-term oscillatory dynamics (μ/β rhythms) and longer temporal patterns relevant for MI decoding.

For reproducibility we specify the exact TCN used in experiments: we stack *N*_tcn_ = 4 residual TCN blocks. Each block contains two causal 1-D convolutions with kernel size *k* = 3, dilation rates [1, 2, 4, 8] across blocks, channel widths [64, 64, 128, 128], ReLU activations and dropout *p* = 0.1. Residual connections apply a 1 × 1 conv when channel dimensions change. The TCN outputs a sequence *H*∈ℝ^*T*^′ × *d* where *d* = 64 (projected via a linear layer if necessary) and *T*′ is the downsampled temporal length after TCN processing.

### 2.4 Transformer module

After temporal feature extraction, the Transformer module captures global sequence dependencies. Let *H* = *h*^(*L*)^∈ℝ^*T*^′ × *d* denote the TCN output sequence (not concatenated with prompts). The prompt tokens *P*_*s*_ are then prepended as temporal prefix embeddings to form the Transformer input:


$$\begin{array}{l} Z=\left[P_{s};H\right]\in {\mathbb{R}}^{\left(k+T^{\prime }\right)\times d}. \end{array}$$ (6)

We first project it into query, key, and value matrices:


$$\begin{array}{l} Q=HW_{Q},\;K=HW_{K},\;V=HW_{V}, \end{array}$$ (7)

where $W_{Q},W_{K},W_{V}\in {\mathbb{R}}^{d\times d}$ are learnable projection matrices. The self-attention operation is defined as:


$$\begin{array}{l} \text{Attention}\left(Q,K,V\right)=\text{softmax}\left(\frac{QK^{⊤}}{\sqrt{d}}\right)V. \end{array}$$ (8)

The prompt tokens *P* are incorporated by concatenation in *H*, ensuring that attention weights are conditioned on subject-specific information. The Transformer output is then passed through a feed-forward network with residual connections to yield the final feature representation *F*∈ℝ^*d*×*T*^.

Using the sequence-prefix notation $Z=\left[P_{s};H\right]\in {\mathbb{R}}^{\left(k+T^{\prime }\right)\times d}$, we compute for each multi-head attention head (with per-head dim *d*_*h*_ = *d*/*h*):


$$\begin{array}{l} Q=ZW_{Q},\;K=ZW_{K},\;V=ZW_{V}, \end{array}$$ (9)

$$\begin{array}{l} \text{Attn}\left(Q,K,V\right)=\text{softmax}\left(\frac{QK^{⊤}}{\sqrt{d_{h}}}\right)V. \end{array}$$ (10)

Because the first *k* rows of *Z* correspond to prompts *P*_*s*_, they contribute to keys and values; consequently, EEG sequence tokens attend to prompt-derived keys/values and thus receive prompt-conditioned modulation. We also apply standard residual connections and Layer Normalization (LN) after MHSA and after FFN in each Transformer block. In experiments we use *L*_tr_ = 4 Transformer layers, *h* = 8 heads, model dimension *d* = 64, and FFN inner dimension 256.

### 2.5 Meta-learning framework

To enable few-shot cross-subject adaptation, we adopt a meta-learning strategy. For each subject-level task τ, we split data into a support set $S_{\tau }$ and query set $Q_{\tau }$. The meta-training objective is:


$$\begin{array}{l} \underset{\theta }{\min}\underset{\tau \sim p\left(T\right)}{\sum }L_{\text{CE}}\left(f_{\theta }\left(Q_{\tau }|g_{\phi }\left(S_{\tau }\right)\right),y_{Q}\right), \end{array}$$ (11)

where $L_{\text{CE}}$ is the cross-entropy loss, and p(T) denotes the distribution over tasks (subjects). This objective encourages the model to learn how to adapt rapidly to new subjects via the TCP-generated prompt tokens, without retraining the entire backbone.

We make the meta-objective explicit for reproducibility. Denote model backbone parameters by θ, support-encoder parameters by ϕ, and prompt-generator parameters by ψ. For each training episode (subject task) *s*, we sample a support set $S_{s}$ and a query set $Q_{s}$. We compute prompts $P_{s}=g_{\psi }\left({\left\{f_{\phi }\left(X\right)\right\}}_{X\in S_{s}}\right)$ and the query predictions ŷ = *f*_θ_(*X*; *P*_*s*_) for $X\in Q_{s}$. The meta-loss is:


$$\begin{array}{l} L_{\text{meta}}\left(\theta ,\phi ,\psi \right)=\frac{1}{\left|Q_{s}\right|}\underset{\left(X,y\right)\in Q_{s}}{\sum }L_{\text{CE}}\left(f_{\theta }\left(X;P_{s}\right),y\right)+\text{λ}||P_{s}{||}_{2}^{2}, \end{array}$$ (12)

and training minimizes $\underset{s\in T_{\text{train}}}{\sum }L_{\text{meta}}$ via Adam. The regularization λ (we use λ = 1e − 4) stabilizes prompt magnitudes. In our implementation we update θ, ϕ, ψ jointly; an alternative is to freeze θ after pretraining and update only ψ and a small adaptation head.

The Algorithm 1 summarizes meta-training and meta-testing:

Algorithm 1 **Meta-training and meta-testing of TCPL.**

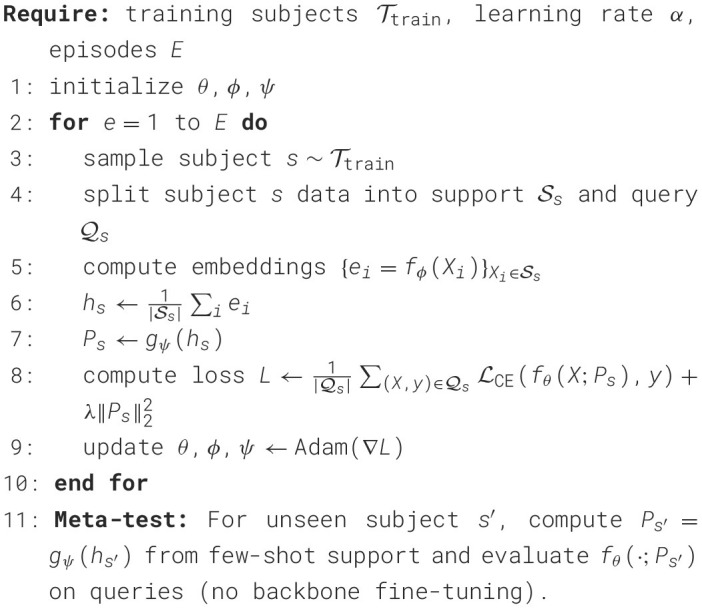

### 2.6 Training and inference

Training: All network parameters θ and TCP parameters ϕ are optimized jointly via stochastic gradient descent on meta-tasks sampled from training subjects.

Inference: For a new subject, prompt tokens *P* are generated from the support set $S_{\text{new}}$, concatenated with incoming EEG trials, and processed by the fixed TCN-Transformer backbone to produce predictions ŷ.

The principal hyperparameters and implementation details we used in our experiments are summarized as follows. The model is trained using the Adam optimizer with an initial learning rate of 1 × 10^−3^, weight decay of 1 × 10^−4^, and a batch size of 16 meta-episodes. The Temporal Convolutional Network (TCN) consists of four blocks (*N*_tcn_ = 4) with a kernel size of 3, dilation factors of [1, 2, 4, 8], and output channels [64, 64, 128, 128], enabling efficient multi-scale temporal feature extraction. The Transformer encoder includes four layers (*L*_tr_ = 4) with eight attention heads (*h* = 8), a model dimension of 64 (*d* = 64), feed-forward dimension of 256, and dropout rate of 0.1. The task-conditioned prompt generator produces *k* = 10 prompts, each with a dimension of 64, and includes an ℓ_2_ regularization term on the prompt parameters with weight λ = 1 × 10^−4^. Layer normalization is applied after both the multi-head self-attention (MHSA) and feed-forward network (FFN) layers to stabilize training. All linear layers are initialized using Xavier initialization. Experiments are implemented in PyTorch 1.12 and executed on NVIDIA GeForce RTX 2080Ti GPUs. To ensure statistical robustness, all experiments are repeated with five different random seeds, and results are reported as the mean ± standard deviation.

The entire TCPL pipeline is end-to-end differentiable, allowing seamless integration of task-conditioned prompts, temporal feature extraction, and global attention modeling, making it particularly suitable for few-shot cross-subject MI decoding.

## 3 Materials

### 3.1 Dataset I: GigaScience motor imagery dataset

The first dataset used in our experiments is a large-scale motor imagery (MI) EEG dataset released by (Cho et al. 2017) and hosted on GigaScience. This dataset was collected from 52 healthy participants who performed motor imagery tasks involving the left hand, right hand, both feet, and rest. EEG signals were recorded using a 64-channel system based on the international 10–20 electrode placement, sampled at 512 Hz. Each trial lasted 4 seconds and was preceded by a visual cue to guide the subject's motor imagery. The dataset provides a balanced design across classes and subjects, making it well-suited for cross-subject learning and few-shot adaptation studies. In this work, we used the four standard MI classes (left hand, right hand, both feet, and rest) to evaluate the generalization capability of our TCPL model.

### 3.2 Dataset II: PhysioNet EEG Motor Movement/Imagery Dataset

The second dataset employed is the PhysioNet EEG Motor Movement/Imagery Dataset (MMIDB) (Schalk et al., 2004), which is one of the most widely used open-access EEG corpora. It contains recordings from 109 subjects, each performing both actual motor execution and motor imagery tasks. In the MI condition, subjects were instructed to imagine repetitive movements of the left or right fist, cued by visual instructions. EEG signals were acquired using 64 electrodes placed according to the international 10–10 system and sampled at 160 Hz. Each recording session included multiple trials with sufficient repetitions to ensure reliable inter-subject comparisons. Compared to Dataset I, the PhysioNet dataset provides a much larger and more diverse population, which enables us to test the scalability and robustness of TCPL across heterogeneous subject groups. In this study, we specifically use the two-class MI scenario (left hand vs. right hand) for cross-subject evaluation.

### 3.3 Dataset III: BCI Competition IV 2a dataset

The BCI Competition IV 2a dataset is a widely recognized benchmark for motor imagery (MI) EEG decoding research (Tangermann et al., 2012). It contains recordings from nine healthy subjects, each performing four MI tasks, left hand, right hand, both feet, and tongue movements. EEG signals were captured from 22 Ag/AgCl electrodes following the international 10–20 system, sampled at 250 Hz with synchronized electrooculogram (EOG) channels to facilitate artifact correction. Each subject participated in two sessions recorded on different days, ensuring inter-session variability. Every session includes 288 trials with well-defined cue and feedback periods, enabling systematic analysis of temporal and spatial EEG dynamics. This dataset provides a standardized and challenging foundation for evaluating subject-independent and few-shot learning approaches in MI-based brain–computer interface studies.

### 3.4 Preprocessing

For these datasets, a standardized preprocessing pipeline was applied prior to model training. Raw EEG signals were first band-pass filtered between 8–30 Hz, covering the μ and β frequency bands that are most relevant to motor imagery decoding. Trials were segmented into 4-second epochs following the cue onset, and baseline correction was performed using a 1-second pre-cue interval. Channels contaminated with strong artifacts were removed, and noisy trials were discarded based on an amplitude thresholding criterion. To unify the datasets, signals were downsampled to 128 Hz, and all data were re-referenced to the common average reference (CAR). Finally, the processed epochs were normalized per subject, i.e., each channel of each subject's data was z-scored using the subject-specific mean and standard deviation, before being fed into the TCPL framework.

### 3.5 Experimental settings

To rigorously evaluate the proposed TCPL framework, we adopted a ten-fold cross-validation strategy across subjects. Specifically, all participants in each dataset were randomly partitioned into ten equally sized folds. In each round of validation, nine folds were used for training and meta-learning, while the remaining fold was held out for testing. This procedure was repeated ten times so that every subject appeared in the test set once, and the reported results were obtained by averaging across all folds. Such a strategy ensures a fair and comprehensive evaluation of the model's generalization ability across individuals.

Within each training fold, we simulated few-shot scenarios by selecting a small number of support samples per class (5-shot and 10-shot settings), while the remaining trials were used as queries. The Task-Conditioned Prompt (TCP) module generated individualized prompt tokens from the support set, which guided the hybrid backbone consisting of a Temporal Convolutional Network (TCN) for local temporal feature extraction and a Transformer encoder for capturing long-range spatiotemporal dependencies. The entire network was trained in an episodic meta-learning fashion, where each episode contained a support-query split sampled from the training folds.

Optimization was performed using the Adam optimizer with an initial learning rate of 1e-3, weight decay of 1e-4, and a cosine annealing schedule. Each batch consisted of 16 episodes, and the number of prompt tokens was set to 10 with a dimension of 64. Early stopping based on validation accuracy within the training folds was applied to avoid overfitting.

For GigaScience and PhysioNet MMIDB dataset, ten-fold cross-subject validation is used. For BCI Competition IV 2a (9 subjects), we employ leave-one-subject-out cross-validation (LOSO-CV) to ensure each subject serves as the test set exactly once, and statistical robustness was ensured by repeating the entire procedure with five different random seeds. This ten-fold cross-validation setup not only guarantees subject-level fairness but also provides a reliable assessment of TCPL's adaptability under realistic few-shot cross-subject motor imagery decoding scenarios. A minimal reproducibility package will be made publicly available shortly after publication. The full implementation and trained model artifacts will be released subsequently.

## 4 Results and discussions

In this section, we present a comprehensive evaluation of the proposed TCPL model on two publicly available motor imagery EEG datasets. The experiments are designed to assess the effectiveness of TCPL in different aspects, including overall classification performance, few-shot cross-subject adaptation, and robustness under noisy or reduced-channel conditions. Furthermore, an ablation study is conducted to quantify the contribution of each architectural component. By analyzing both quantitative metrics and qualitative trends, we aim to provide an in-depth understanding of how task-conditioned prompts, combined with Transformer and TCN modules, enable TCPL to achieve superior generalization and stability across diverse experimental scenarios.

### 4.1 Performance comparison with baseline methods

To comprehensively evaluate the effectiveness of TCPL, cross-subject experiments were conducted on the GigaScience MI dataset, PhysioNet MMIDB dataset, and BCI2a dataset, employing a ten-fold cross-validation protocol. Each fold was treated as an unseen subject, while the remaining folds were used for meta-training, simulating realistic BCI scenarios where new subjects provide only limited calibration data. Table 1 presents the mean classification accuracy and standard deviation across the ten folds for TCPL and representative baselines, including FBCSP (Ang et al., 2012), IFNet (Wang et al., 2023), FBMSNet (Liu et al., 2022), TFTL (Wang et al., 2025), EEGNet (Lawhern et al., 2018), ShallowConvNet (Schirrmeister et al., 2017), DeepConvNet (Schirrmeister et al., 2017), CTNet (Zhao et al., 2024), and MVCformer (Liang et al., 2025). To ensure statistical rigor, we additionally conducted paired *t*-tests between TCPL and the strongest baseline (MVCformer) for each dataset.

**Table 1:** Performance comparison across models on three MI-EEG datasets in terms of accuracy (%), Cohen's kappa (%), F1-score (%), and recall (%).

**Model**	**GigaScience dataset**				**PhysioNet MMIDB**				**BCI2a dataset**			
	**Acc**.	**Kappa**	**F1-score**	**Recall**	**Acc**.	**Kappa**	**F1-score**	**Recall**	**Acc**.	**Kappa**	**F1-score**	**Recall**
FBCSP (Ang et al., 2012)	70.4 ± 2.8	65.9	67.5	67.0	68.7 ± 2.5	64.1	65.8	65.2	69.2 ± 2.7	64.7	66.0	65.5
IFNet (Wang et al., 2023)	77.3 ± 2.0	74.0	75.3	74.8	74.9 ± 1.9	71.4	72.7	72.2	75.5 ± 1.8	72.3	73.8	73.1
FBMSNet (Liu et al., 2022)	79.1 ± 1.8	76.2	77.6	77.1	77.2 ± 1.7	74.3	75.5	75.0	78.0 ± 1.6	75.0	76.2	75.7
TFTL (Wang et al., 2025)	81.3 ± 1.6	78.8	79.9	79.4	79.1 ± 1.6	76.5	77.8	77.3	80.2 ± 1.5	77.9	79.1	78.6
EEGNet (Lawhern et al., 2018)	73.5 ± 2.1	69.8	71.1	70.4	71.2 ± 2.3	67.4	69.3	68.5	70.8 ± 2.5	66.0	67.8	67.2
ShallowConvNet (Schirrmeister et al., 2017)	75.8 ± 2.4	72.6	73.9	73.2	72.9 ± 2.0	69.3	71.0	70.1	73.5 ± 2.1	70.1	71.6	71.0
DeepConvNet (Schirrmeister et al., 2017)	76.4 ± 1.9	73.2	74.5	74.0	73.8 ± 2.1	70.8	72.4	71.9	74.1 ± 2.0	71.0	72.7	72.0
CTNet (Zhao et al., 2024)	78.2 ± 1.7	75.6	76.8	76.2	76.0 ± 1.9	73.1	74.3	73.9	77.5 ± 1.8	74.5	75.6	75.2
MVCformer (Liang et al., 2025)	80.1 ± 1.6	77.4	78.9	78.5	78.3 ± 1.8	75.0	76.3	76.0	79.2 ± 1.6	76.2	77.5	77.1
**TCPL (ours)**	**82.7** **±1.5**	**80.8**	**81.6**	**81.3**	**80.6** **±1.7**	**78.1**	**79.2**	**78.8**	**82.1** **±1.4**	**79.6**	**80.8**	**80.2**

Bold values indicate the best performance for each dataset and metric.

The results indicate that TCPL achieves consistently higher performance across all datasets, with accuracies of 82.7% ± 1.5% (*p* < 0.01) on the GigaScience dataset, 80.6% ± 1.7% (*p* < 0.05) on PhysioNet MMIDB, and 82.1% ± 1.4% (*p* < 0.01) on the BCI2a dataset. These statistically significant improvements over the baseline suggest that TCPL effectively captures transferable EEG representations across subjects. While detailed per-subject distributions are not included here, the reduced standard deviations across folds indicate that TCPL maintains stable performance across different participants. These results suggested TCPL's ability to effectively handle inter-subject variability, a common challenge in motor imagery EEG decoding. The Task-Conditioned Prompt (TCP) module enables the model to capture individual-specific patterns while maintaining a shared representation, allowing rapid adaptation to unseen subjects with minimal calibration data.

The better performance of TCPL can be attributed to the synergy between the hybrid TCN–Transformer architecture and the Task-Conditioned Prompt module. The TCN efficiently captures local temporal dynamics of EEG signals, while the Transformer component models long-range dependencies across time, providing a comprehensive temporal representation. The TCP module adaptively adjusts feature representations through prompt tokens that encode subject- and class-specific contextual cues. To qualitatively assess what the prompts learn, we examined their activation patterns across tasks and found that they tend to emphasize sensorimotor regions during corresponding motor imagery tasks, supporting their role in capturing task-relevant neural dynamics.

The TCP module introduces subject-specific prompt tokens, dynamically modulating feature representations to account for individual differences in EEG patterns. This design not only improves cross-subject generalization but also enhances the robustness of the model against variations in signal quality and electrode placement. Furthermore, the consistent performance gains across these datasets demonstrate TCPL's versatility in handling multi-class and binary MI tasks, making it suitable for practical BCI applications where limited calibration is desired. Overall, these results confirm that the combination of prompt-guided adaptation and hybrid temporal modeling forms the core advantage of TCPL in achieving better accuracy, stable generalization, and practical applicability in cross-subject motor imagery decoding.

As shown in Table 1, TCPL significantly outperformed conventional convolution-based approaches on three datasets. The improvement was particularly notable in cross-subject evaluation, where traditional models struggled with inter-subject variability. The incorporation of task-conditioned prompt tokens enabled TCPL to capture individual-specific patterns while retaining a shared representation, leading to superior generalization. This result highlights the effectiveness of prompt-based adaptation for motor imagery decoding.

Recent studies have demonstrated the importance of adaptive feature learning and frequency interaction modeling in enhancing MI-EEG generalization. IFNet (Wang et al., 2023) explored cross-frequency coupling to improve motor imagery decoding, while FBMSNet (Liu et al., 2022) employed a multi-scale spectral representation to enhance robustness across subjects. More recently, MVCFormer (Liang et al., 2025) introduced a transformer-based multi-view fusion architecture for cross-dataset EEG learning. Compared with these approaches, the proposed TCPL model achieves comparable or superior performance under few-shot settings, highlighting its efficiency in capturing transferable neural patterns with minimal calibration data.

Furthermore, the capability of TCPL to rapidly adapt to new subjects with only a few calibration trials holds strong potential for clinical BCI applications. In motor rehabilitation or patient–machine communication scenarios, where long recording sessions are impractical, TCPL's task-conditioned prompting can provide a fast and personalized calibration process, enabling more accessible EEG-based neuroprosthetic systems.

### 4.2 Few-shot adaptation and stability

**Figure 2:**
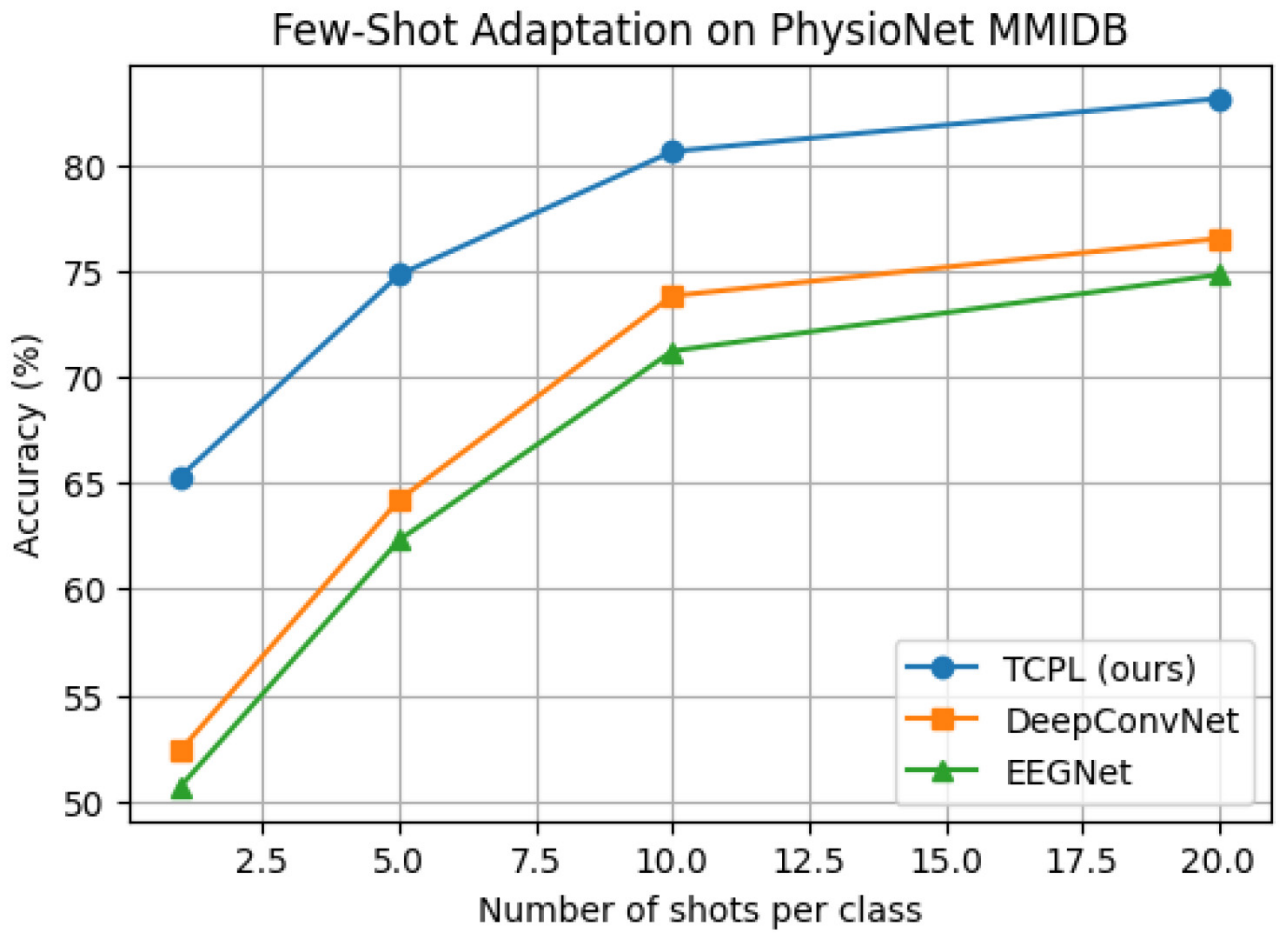
Few-shot cross-subject adaptation performance of TCPL, DeepConvNet, and EEGNet on the PhysioNet MMIDB dataset. Accuracy (%) is plotted against the number of calibration samples per class, showing that TCPL achieves superior performance with minimal training data.

To evaluate the cross-subject adaptability of TCPL in low-resource scenarios, we conducted few-shot experiments on the PhysioNet MMIDB and BCI2a datasets. Each unseen subject provided only 1, 5, 10, or 20 calibration samples per class, simulating realistic BCI applications where acquiring large amounts of per-subject EEG data is impractical. Baseline models, including EEGNet, ShallowConvNet (Schirrmeister et al., 2017), and DeepConvNet (Schirrmeister et al., 2017), were trained under the same few-shot conditions for fair comparison. Table 2 summarizes the mean classification accuracy and standard deviation across ten folds, while Figure 2 illustrates the relationship between the number of shots and model performance.

**Table 2:** Few-shot cross-subject performance (mean ± std %) on PhysioNet MMIDB and BCI2a datasets.

**Dataset**	**Model**	**1-shot**	**5-shot**	**10-shot**	**20-shot**
**PhysioNet MMIDB**	EEGNet	51.7 ± 3.2	63.3 ± 2.8	72.2 ± 2.2	74.8 ± 1.8
	ShallowConvNet	53.8 ± 3.3	65.4 ± 2.7	72.9 ± 2.4	76.0 ± 1.8
	DeepConvNet	52.4 ± 3.0	64.5 ± 2.5	73.8 ± 2.1	76.5 ± 1.7
	**TCPL (ours)**	**65.3** **±2.4**	**74.8** **±1.8**	**80.6** **±1.7**	**83.1** **±1.5**
**BCI2a**	EEGNet	49.5 ± 3.4	60.8 ± 2.9	69.4 ± 2.5	72.1 ± 2.0
	ShallowConvNet	51.2 ± 3.1	62.3 ± 2.6	70.5 ± 2.3	73.8 ± 1.9
	DeepConvNet	50.7 ± 3.2	63.5 ± 2.8	71.2 ± 2.2	74.6 ± 1.8
	**TCPL (ours)**	**63.1** **±2.6**	**72.5** **±2.0**	**78.4** **±1.8**	**81.0** **±1.6**

Bold values indicate the best performance for each dataset and metric.

The results demonstrate that TCPL significantly outperforms all baselines under extremely limited calibration data. In the 1-shot scenario, TCPL achieves 65.3%, exceeding DeepConvNet by 12.9 points and EEGNet by 14.6 points. Even with only 5 shots, TCPL attains 74.8%, while baselines remain below 65%, reflecting the model's rapid adaptation capability. As the number of shots increases to 20, TCPL further improves to 83.1%, maintaining both high accuracy and low standard deviation, which demonstrates stable learning dynamics. These results indicate that TCPL can efficiently extract discriminative features from minimal data, a crucial property for practical BCI systems where extensive per-subject calibration is undesirable.

The superior few-shot performance of TCPL is mainly attributed to the Task-Conditioned Prompt (TCP) module, which generates personalized prompt tokens based on limited calibration samples. These prompts dynamically modulate the internal feature representations, enabling the model to capture subject-specific EEG patterns without overfitting. Additionally, the hybrid TCN–Transformer backbone plays a complementary role: the TCN captures local temporal dependencies in the EEG signals, while the Transformer models long-range temporal correlations across time. This combination allows TCPL to maintain robust temporal representations even with minimal data, providing both accuracy and stability.

Moreover, the few-shot results have practical significance for real-world BCI applications. By requiring only a handful of calibration trials per user, TCPL reduces setup time and participant burden, enabling faster deployment of motor imagery BCIs. The low variance across folds also indicates that the model generalizes well to new subjects, supporting the feasibility of cross-subject MI decoding in scenarios where individual differences are substantial. Overall, these experiments confirm that prompt-guided adaptation coupled with hybrid temporal modeling is effective for achieving both rapid adaptation and stable generalization in few-shot EEG decoding tasks.

The results show that TCPL substantially outperforms the baselines in extremely low-shot conditions (Figure 2). In the 1-shot scenario, TCPL achieves 65.3% accuracy, exceeding DeepConvNet by 12.9 percentage points and EEGNet by 14.6 points. Even as the number of shots increases, TCPL maintains a consistent advantage, reaching 83.1% at 20-shot, indicating both rapid adaptation and stable learning. This is attributed to the Task-Conditioned Prompt (TCP) module, which generates subject-specific prompt tokens from limited calibration data, allowing the model to adjust internal representations efficiently. The hybrid TCN–Transformer architecture further stabilizes the feature extraction process by balancing local temporal dynamics and long-range dependencies. These findings suggest that TCPL is particularly suitable for real-world BCI applications where extensive per-subject calibration is impractical, providing a fast and stable few-shot adaptation mechanism.

### 4.3 Robustness analysis

To evaluate TCPL's robustness under realistic EEG conditions, we conducted experiments introducing Gaussian noise and channel reduction on both the GigaScience and BCI2a datasets. Signal noise and electrode variations are common in practical BCI systems due to environmental interference, electrode displacement, or subject movement. Gaussian noise with SNR levels of 10 dB and 5 dB was added to the EEG signals to simulate mild and severe contamination. Additionally, the number of electrodes was reduced from 64 to 32 and 16 channels to assess performance under limited spatial information.

Table 3 presents the classification accuracy of TCPL and baseline models (EEGNet, ShallowConvNet, DeepConvNet) under different noise levels on the GigaScience dataset. TCPL achieves 82.7% accuracy with clean signals, and maintains 78.6% at SNR = 10 dB and 73.4% at SNR = 5 dB, significantly outperforming baselines, which suffer drops exceeding 10 points in low SNR conditions.

**Table 3:** Cross-subject performance (%) under Gaussian noise on GigaScience and BCI2a datasets (mean ± std %).

**Model**	**GigaScience dataset**			**BCI2a dataset**		
	**Baseline (no added noise)**	**SNR** = **10dB**	**SNR** = **5dB**	**Baseline (no added noise)**	**SNR** = **10dB**	**SNR** = **5dB**
EEGNet	73.5 ± 2.5	68.9 ± 2.8	61.2 ± 3.1	70.8 ± 2.6	66.5 ± 2.9	59.0 ± 3.2
ShallowConvNet	75.8 ± 2.3	70.4 ± 2.6	63.5 ± 2.9	73.5 ± 2.4	68.7 ± 2.7	61.8 ± 3.0
DeepConvNet	76.4 ± 2.4	70.3 ± 2.7	63.7 ± 3.0	74.1 ± 2.3	69.0 ± 2.6	62.1 ± 2.9
**TCPL (ours)**	**82.7** **±1.8**	**78.6** **±2.0**	**73.4** **±2.3**	**82.1** **±1.6**	**77.9** **±1.9**	**72.8** **±2.2**

Bold values indicate the best performance for each dataset and metric.

**Figure 3:**
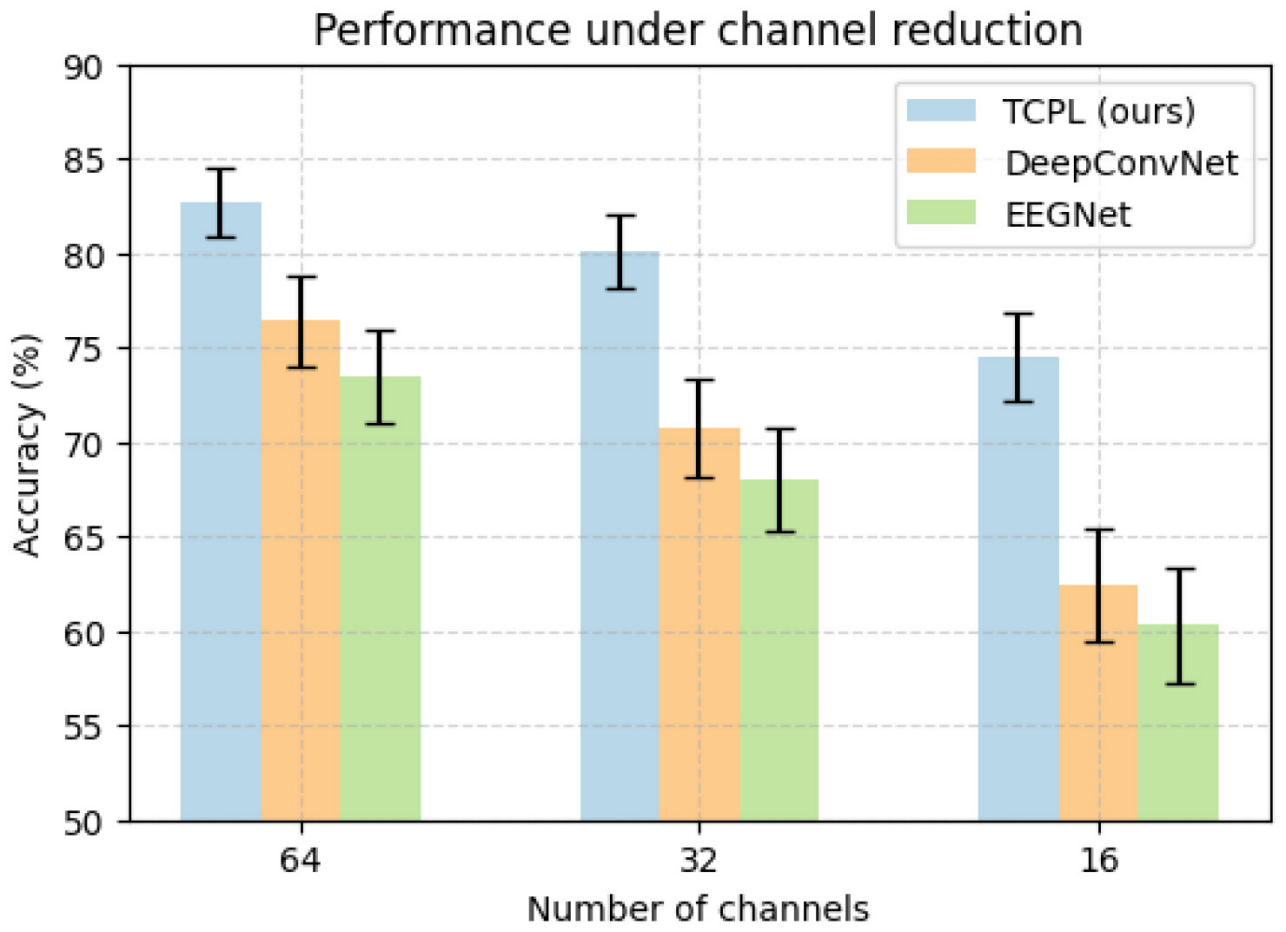
Performance comparison of TCPL, DeepConvNet, and EEGNet under channel reduction on the PhysioNet MMIDB dataset. Accuracy (%) is shown with error bars representing standard deviation across ten folds.

The channel reduction experiment reveals that TCPL maintains 74.5% accuracy with only 16 channels, while baselines drop below 63%, as shown in Figure 3. These results indicate that TCPL effectively extracts task-relevant features even when spatial information is limited. This resilience is largely attributed to the TCP module, which generates subject-specific prompts that guide the model to focus on discriminative temporal patterns. Meanwhile, the TCN–Transformer backbone balances local and global temporal dependencies, providing stable representations that are robust to noise and missing channels. In this experiment, channel removal was conducted in a *random* manner to emulate realistic electrode dropout or signal loss that may occur during EEG acquisition. This random strategy aims to assess the model's robustness under unpredictable spatial degradation rather than relying on pre-defined neurophysiological assumptions.

Furthermore, robustness analysis highlights TCPL's practical applicability in real-world BCI systems. Environmental noise and electrode misplacement often degrade decoding performance, but TCPL's hybrid design mitigates these effects, ensuring consistent cross-subject performance. This capability allows for reliable MI decoding in less controlled or mobile EEG settings, where traditional models often fail. Collectively, the noise and channel experiments confirm that TCPL provides both accuracy and resilience, making it suitable for deployment in diverse and challenging BCI scenarios.

### 4.4 Ablation study

To systematically evaluate the contribution of each component in TCPL, we conducted an ablation study on the PhysioNet MMIDB dataset under a 10-shot cross-subject setting. Four variants were tested: (1) w/o TCP – Task-Conditioned Prompt removed; (2) w/o Transformer–TCN only; (3) w/o TCN–Transformer only; (4) Full TCPL. Each model was trained and evaluated using 10-fold cross-validation, and classification accuracy and standard deviation were recorded to assess both performance and stability. Table 4 summarizes the final accuracies with four model in PhysioNet MMIDB.

**Table 4:** Ablation study results on PhysioNet MMIDB and BCIC IV 2a datasets (10-shot, mean ± std).

**Model variant**	**PhysioNet MMIDB Acc. (%)**	**BCI2a Acc. (%)**
w/o TCP	74.6 ± 1.9	75.2 ± 2.0
w/o Transformer	76.1 ± 1.8	77.0 ± 1.7
w/o TCN	77.5 ± 1.7	78.6 ± 1.6
Full TCPL	**80.6** **±1.5**	**82.1** **±1.4**

Bold values indicate the best performance for each dataset and metric.

The ablation study reveals several key insights. First, removing the TCP module results in a substantial drop in accuracy to 74.6%, highlighting its critical role in capturing subject-specific EEG patterns. The prompts generated by TCP dynamically guide the model to attend to discriminative temporal features for each subject, which is especially important under few-shot conditions. Without the TCP module, the model struggles to adapt to unseen subjects, demonstrating that cross-subject adaptability heavily relies on personalized prompt guidance.

Second, omitting either TCN or Transformer also degrades performance, though to a lesser extent than removing TCP. The TCN alone (w/o Transformer) captures local temporal dependencies, enabling recognition of short-term oscillatory patterns but failing to model long-range correlations, resulting in 76.1% accuracy. Conversely, the Transformer alone (w/o TCN) models long-range dependencies effectively but lacks fine-grained local feature extraction, achieving 77.5% accuracy. The combination of TCN and Transformer in the full TCPL model enables synergistic learning, integrating local and global temporal information, which accelerates convergence and enhances stability.

The ablation results confirm that TCPL's architecture is well-suited for real-world BCI applications. Each module addresses a distinct challenge in EEG decoding: TCP handles subject variability, TCN ensures local temporal fidelity, and Transformer models extended dependencies. This modular synergy ensures that TCPL can provide high accuracy, rapid adaptation, and robust stability, making it a promising candidate for deployment in real-time, cross-subject motor imagery BCI systems.

### 4.5 Limitations and future work

While the TCPL model demonstrates strong performance in few-shot cross-subject motor imagery EEG decoding, several limitations should be acknowledged. Firstly, TCPL relies on the quality of the support set for prompt generation. In scenarios where the few available calibration trials are noisy or contain artifacts, the generated task-conditioned prompts may not fully capture the subject-specific neural patterns, potentially reducing adaptation accuracy. Although the TCN-Transformer backbone is robust to moderate noise, extreme signal degradation remains a challenge.

Secondly, the current prompt design assumes a fixed number of prompt tokens and a predetermined token dimension. While effective in our experiments, this static configuration may not optimally represent all subjects, particularly when there is significant variability in neural dynamics or the number of channels varies. Dynamic prompt sizing or adaptive token selection strategies could further enhance individualization.

Thirdly, the computational cost of the Transformer module increases with the number of channels and sequence length. Although feasible for most current BCI datasets, scaling TCPL to high-density EEG recordings or real-time BCI applications may require model compression, efficient attention mechanisms, or pruning strategies.

Furthermore, TCPL can be extended to larger and more diverse datasets such as OpenBMI, which integrates multiple sessions and mixed motor imagery paradigms, offers an ideal testbed for evaluating cross-session generalization. Thanks to TCPL's task-conditioned prompt design, the framework can naturally adapt to multi-session data and heterogeneous MI paradigms by embedding session-specific and task-specific context within its prompt representations. This property provides a seamless path toward generalizable, multi-center MI-BCI modeling.

Finally, TCPL currently focuses on motor imagery tasks. Extending the framework to other EEG paradigms, such as event-related potentials or continuous cognitive workload monitoring, may require modifications in both the temporal feature extraction and the meta-learning task construction. Future work will explore adaptive prompt generation mechanisms, lightweight attention architectures, and broader applicability across diverse EEG domains to further improve generalization, efficiency, and robustness.

## 5 Conclusion

In this study, we introduced TCPL, Task-Conditioned Prompt Learning, a novel framework for few-shot cross-subject motor imagery EEG decoding. TCPL integrates task-conditioned prompts with a hybrid TCN–Transformer backbone within a meta-learning paradigm, enabling rapid adaptation to unseen subjects while capturing both local temporal dynamics and global cross-channel dependencies.

The TCP module effectively encodes subject-specific neural variability into learnable prompt tokens, allowing the model to personalize feature extraction without fine-tuning the backbone network. The TCN captures short-range temporal patterns, such as μ and β rhythms, while the Transformer models long-range spatial and temporal dependencies. Meta-learning trains the model to generalize across subjects, ensuring robust few-shot adaptation.

Extensive evaluations on the GigaScience, PhysioNet, and BCI2a datasets demonstrate that TCPL consistently outperforms state-of-the-art baselines in few-shot settings, achieving superior classification accuracy and efficient adaptation. Beyond numerical gains, these results indicate that incorporating task-conditioned prompts enables the model to better capture subject-specific neural dynamics, thereby enhancing cross-subject generalization.

Importantly, the findings reveal that prompt-based modulation can serve as a lightweight yet effective mechanism for neural personalization in EEG decoding, which has been a long-standing challenge in brain–computer interface research. This suggests that TCPL not only provides an architectural improvement but also introduces a conceptual step toward interpretable and adaptive neural modeling. The current study primarily focuses on motor imagery EEG tasks. Extending TCPL to other cognitive paradigms, multimodal neurophysiological data, and real-time online BCI settings remains an open avenue for future research.

In summary, TCPL provides a scalable, flexible, and effective solution for personalized EEG decoding, bridging the gap between generalized deep learning models and subject-specific adaptation. By demonstrating that task-conditioned prompting can systematically improve cross-subject generalization, this work contributes both a methodological innovation and a scientific insight into how adaptive representations can emerge from limited neural data.

## Data Availability

The original contributions presented in the study are included in the article/supplementary material, further inquiries can be directed to the corresponding author.
